# Supplementary material for: Yiai Fuzheng decoction inhibits triple-negative breast cancer by remodeling the immune microenvironment
Source: Front Immunol. 2025 Sep 30;16:1615631. doi: 10.3389/fimmu.2025.1615631 (PMC12518410; doi:10.3389/fimmu.2025.1615631)
Supplement: Supplementary file 2 [file DataSheet1.zip › Supplementary File 2/Treatment/C_vs_D/02_PCA/All_Groups/PCA_Screeplot.pdf]

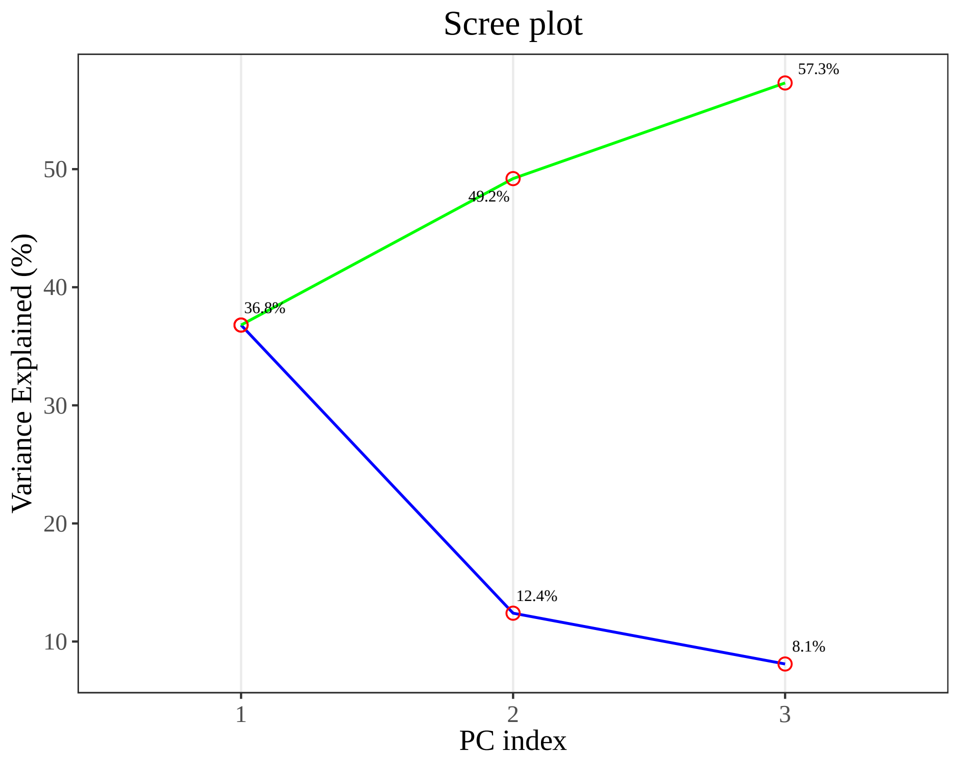
Scree plot
Variance Explained (%)
PC index
10
20
30
40
50
1
2
3
36.8%
49.2%
57.3%
12.4%
8.1%
